# An *ANKRD26* nonsense somatic mutation in a female with epidermodysplasia verruciformis (Tree Man Syndrome)

**DOI:** 10.1002/ccr3.1595

**Published:** 2018-06-05

**Authors:** K. M. Furkan Uddin, Robed Amin, Sabbiha Nadia Majumder, Mohammad Abdul Aleem, Atikur Rahaman, Nushrat Jahan Dity, M. D. Abdul Baqui, Hosneara Akter, Muhammad Mizanur Rahman, Marc Woodbury‐Smith, Stephen Scherer, Mohammed Uddin

**Affiliations:** ^1^ Holy Family Red Crescent Medical College Dhaka Bangladesh; ^2^ NeuroGen Technologies Ltd. Dhaka Bangladesh; ^3^ Department of Medicine Dhaka Medical College Dhaka Bangladesh; ^4^ International Centre for Diarrhoeal Disease Research, Bangladesh (ICDDR,B) Dhaka Bangladesh; ^5^ Department of Pediatrics Bangabandhu Sheikh Mujib Medical University (BSMMU) Dhaka Bangladesh; ^6^ Institute of Neuroscience Newcastle University Newcastle upon Tyne UK; ^7^ The Centre for Applied Genomics The Hospital for Sick Children Toronto ON Canada; ^8^ Program in Genetics and Genome Biology (GGB) The Hospital for Sick Children Toronto ON Canada; ^9^ Department of Molecular Genetics University of Toronto Toronto ON Canada; ^10^ McLaughlin Centre University of Toronto Toronto ON Canada; ^11^ College of Medicine Mohammed Bin Rashid University of Medicine and Health Sciences Dubai UAE

**Keywords:** epidermodysplasia verruciformis, nonsense mutation, somatic mutation, whole genome sequencing

## Abstract

Epidermodysplasia verruciformis (EV) is an extremely rare hereditary skin disease characterized by an abnormal susceptibility to the human papilloma virus (HPV) with an increased risk of cutaneous malignancy. Here we report the first female severe EV case in Bangladesh, a 10‐year‐old girl with a nonsense somatic mutation impacting *ANKRD26* gene.

## 1 BACKGROUND

Epidermodysplasia verruciformis (EV) is a rare disease of the skin, characterized by abnormal susceptibility to certain types of the human papilloma virus (HPV). In the most severe form, an uncontrolled proliferation of keratinocytes results in bark‐like tissue outgrowths, principally on the hands and feet.^1^, ^2^ A range of dermatological manifestations are reported: in addition to the most striking bark‐like lesions (which, along with its almost male exclusivity, coined the term “tree man syndrome”), pigmented macules and wart like lesions may also be observed, and malignant skin conditions may ensue.^1^, ^3^ Remarkably, these same viruses are believed ubiquitous and asymptomatic in the population‐at‐large, and the etiology in cases reported so far points toward a specific functional deficiency in HPV‐associated cell‐mediated immunity with a genetic underpinning. An autosomal recessive pattern of inheritance predominates and homozygous mutations in the two genes *EVER1(TMC6)* and *EVER2(TMC8)* have been described in the majority (~75%) of cases.^2^, ^4^, ^5^ These genes encode integral membrane proteins, which are likely to function as modifiers of ion transporters or channels and to be involved in signal transduction.^6^, ^7^ They are highly expressed in lymphocytes, but the mechanism whereby mutations result in a defective immune response is not known.

## 2 METHODS

We have collected patient written consent to collect blood and bark‐like tissue for the purpose of research. DNA was extracted from blood using ReliaPrep Blood gDNA Miniprep System and GeneJET Genomic DNA Purification Kit, respectively, following standard procedure. The concentration was 117 and 34 ng/μL for blood and tree bark‐like tissue, respectively. We have conducted whole genome sequencing using illumina HiseqX system. Briefly, about 1 μg of genomic DNA samples with OD260‐280 between 1.8 and 2.0 was submitted to Toronto Centre for Applied Genomics (TCAG) for whole genome sequencing. DNA quantification was performed using Qubit High Sensitivity Assay and checked sample purity using Nanodrop OD260/280 ratio. One hundred nanograms of DNA was used as starting material for library preparation using the Illumina TruSeq DNA PCR‐Free Library Prep Kit following standard manufacturer’s recommended protocol. Five hundred nanograms of DNA was fragmented to 350 bp on average using Covaris LE220 sonication instrument and fragmented DNA was end‐repaired subsequently. A‐tailed and indexed TruSeq Illumina adapters with overhang‐T were ligated to the DNA fragments, and the constructed libraries were validated on a Bioanalyzer DNA High Sensitivity chip to detect the fragment size and the absence of primer dimers. In addition, library was quantified by qPCR using Kapa Library Quantification Illumina/ABI Prism Kit protocol (KAPA Biosystems). Next, the validated libraries were pooled in equimolar quantities and paired‐end sequenced on an Illumina HiSeq X platform following Illumina’s recommended protocol to generate paired‐end reads of 150 bases in length.

We have used illumina provided software for alignment and mutation (single neulcleotide variants and copy number variants) detection. To identify pathogenic missense mutations, we have applied a strict criteria^8^, ^9^ SIFT ≤ 0.05, Polyphen2 ≥ 0.95, CADD≥15, Mutation Assessor score ≥2 and PhyloP ≥2.4). To identify somatic mutation, alternative allele frequency percentage 10%‐40% was considered with at least 10 supporting reads. To validate clinically relevant mutations, we have applied standard Sanger sequencing technique. Histopathological analysis was applied using standard protocols on the resected tree bark‐like tissue slice.

## 3 RESULTS

### 3.1 Clinical report

Here we describe the first female (age 10 years) with a confirmed diagnosis of severe epidermodysplasia verruciformis in Bangladesh and probably is the first case with the mutation in *ANRD26*. The research was conducted with the approval from the patient’s family, who provided written consent for the preparation of this case report. All clinical information was based on detailed examination by several physicians experienced in the diagnosis and treatment of skin disorders.

**Figure 1:**
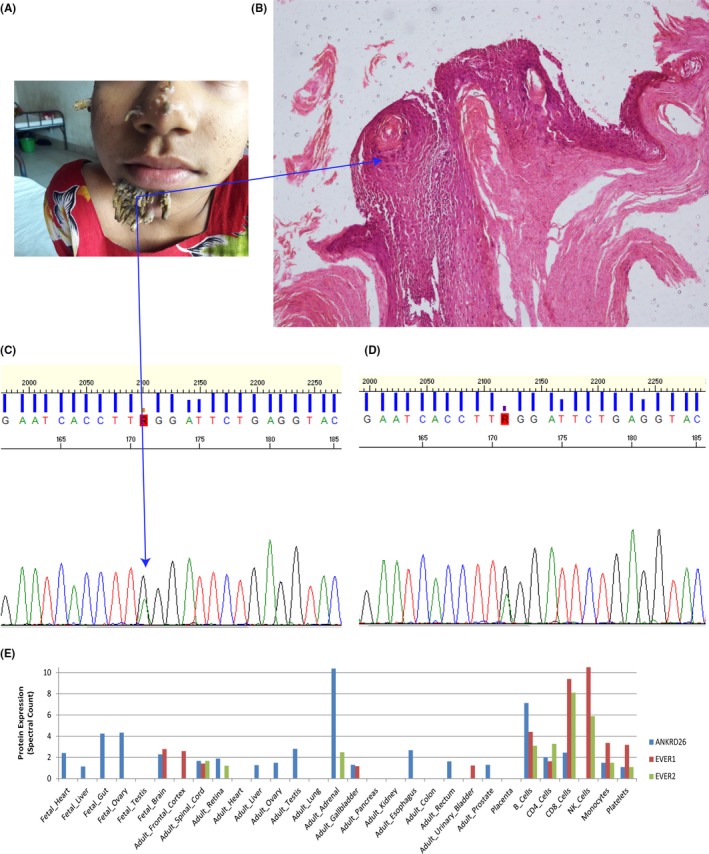
Histology and molecular profile of a female epidermodysplasia verruciformis case; A, presurgical status of nontender papulonodularor tree bark‐like lesion observed in multiple facial spots; B, histology report from immunohistochemistry on the surgically resected bark‐like lesion shows large cytoplasm characteristic to HPV and parakeratosis; C, Sanger sequencing chromatogram for DNA extracted from bark‐like tissue and D, blood showing consistently the differential pick of the mosaic stop gain mutation (T allele). E, Protein expression profile of *ANKRD26*,*EVER1* and *EVER2* from 30 developmental (prenatal to adult) human tissues. The *y*‐axis is the gene protein spectral count for each tissue

The patient is from Dhaka, Bangladesh, where four unrelated male patients have previously been described with the same, severe skin manifestations. She was 12 months old when first observed to have a skin rash. Prior to this, she was otherwise physically healthy and was born following an uncomplicated pregnancy and delivery and with all growth and developmental milestones appropriate for her age. Her nonconsanguineous parents are also physically healthy with no dermatological conditions. She has no siblings. The rash was initially located on the extensor surface of her right thigh and knee, and flexor surfaces of her elbows. These painless, nonitchy and nondischarging rashes subsequently coalesced to form hyperpigmented areas. Painful, itchy warty lesions began at the age of 6 years on the right side of her face and then spread to involve her chin, nose, and both ear lobules simultaneously (Figure 1A). On examination, multiple horn‐like projections were identified, each about 2‐3 cm in size and conical in shape, with a further smaller lesion on her thigh (Figure 1A). Additionally, multiple, nontender papulonodular lesions were identified, involving the flexor surfaces of both upper limbs and extensor surface of her right thigh and knee. Sensation was retained in all lesions examined. Surgical intervention successfully removed the larger bark‐like lesions, but unfortunately within 2 weeks the lesions had started to return.

Laboratory examination was unremarkable: her complete blood cell count and biochemical blood parameters were all in the normal range. Viral markers for hepatitis B and C were also negative, and chest X‐ray was normal. Histology of excised tissue from her face showed the presence of swollen cytoplasm revealing the suggestive presence of HPV and parakeratosis (Figure 1B).

### 3.2 Mutation detection

We carried out whole genome sequencing (WGS) at TCAG (Toronto, Canada), using the Illumina Hiseq X system (applying DNA PCR‐Free Library Prep Kit) with an average read depth of 40.9X. We initially screened for mutations in *EVER1* and *EVER2*. No rare (MAF <1%), exonic mutations that were predicted damaging were found in these genes. We also used WGS data to examine for the presence of structural variants, but none that were rare, exon impacting and deemed clinically significant were identified. Our wider search for rare loss of function (LoF: nonsense, splice site and frameshift) and damaging missense mutations identified a rare somatic stop gain mutation in exon 11 of *ANKRD26* with an allele frequency of 40%. This mutation is not listed in the ExAC server. This mutation impacts transcription of 22 downstream exons. We validated the mutation using Sanger sequencing where the differential allele trace supports the presence of high‐frequency somatic mutation. We also confirmed the presence of the somatic mutation in DNA, extracted from excised bark‐like tissue (Figure 1C). Unfortunately we did not have parental blood to confirm the transmission of this mutation.

## 4 DISCUSSION AND CONCLUSION

*ANKRD26* is an ancestral gene for the primate‐specific POTE gene family. It is associated with the inner cell membrane, interacting with signaling proteins, but its functional role has not been fully elaborated. mRNA expression profiles reveal abundance in a variety of tissues, notably lymphocytes, brain, and the pituitary gland. Mutations impacting the 5′UTR have been strongly associated with an inherited form of thrombocytopenia, with some individuals also developing myeloid malignancies. None of the cases described in the literature with thrombocytopenia and/or myeloid malignancies have skin conditions. The phenotypic consequences of mutations elsewhere in the gene have not previously been described.

There are some similarities between *ANKRD26* and the *EVER* genes that supports its primary role for the skin condition described. For example, all are highly expressed in lymphocytes, but are not expressed in skin. This points to a principal role for immune‐mediated cell proliferation in association with HPV for all three genes. It is possible that EVER genes and *ANKRD26* impact pathways that are highly active in skin. Both *ANKRD26* and the *EVER* genes are also functionally related to cell signaling. Protein expression analysis on 30 developmental human tissues (from 6 prenatal to 24 adult)^10^, ^11^ revealed strikingly similar expression pattern of *ANKRD26* with *EVER1* and *EVER2* genes (Figure 1E). All three genes are highly expressed in hematopoietic cells, specifically in B‐cells, CD4‐cells, and CD8‐cells compared with other tissues. Although ~75% of cases of EV described so far have *EVER* mutations, the remaining cases are of unknown etiology. We believe that it is possible therefore that these and other cases of EV may be associated with mutations in *ANKRD26*.

In summary, therefore, we have described a girl with a severe form of EV who harbors a nonsense mutation in *ANKRD26*. This is the first case of an association between EV and *ANKRD26*, with evidence of pleiotropy in association with the location of the mutation. It remains to be seen whether other cases of EV who do not have *EVER* mutations are also associated with *ANKRD26* mutations.

## AUTHOR’S CONTRIBUTION

MU and SS: conceived the project, wrote the manuscript and helped with analysis. KMFU and RA: collected the sample, conducted experimental analysis and wrote the manuscript. SNM, MAA, MDAB, and MMR: collected phenotypic information. AR, NJD, HA: helped with sequencing analysis; MWS: helped with data analysis and writing the manuscript.

## CONFLICT OF INTEREST

The authors declare no conflict of interest.
